# RT-PCR tests for SARS-CoV-2 processed at a large Italian Hospital and false-negative results among confirmed COVID-19 cases

**DOI:** 10.1017/ice.2020.290

**Published:** 2020-06-11

**Authors:** Francesca Valent, Anna Doimo, Giada Mazzilis, Corrado Pipan

**Affiliations:** Institute of Hygiene and Clinical Epidemiology, University Hospital of Udine, Udine, Italy

*To the Editor*—In Italy, the first autochthonous case of coronavirus disease 2019 (COVID-19) was detected on February 21, 2020. By mid April 2020, >15,000 persons had been infected in the country and >20,000 had died.^1^ More than 1 million upper respiratory specimens were collected through nasopharyngeal or oropharyngeal swabs^2^ for infection confirmation or screening purposes. The proportion of the population with confirmed infection varies across the 20 Italian regions, as does the number of swabs collected per population unit.^2^

Real-time reverse-transcriptase polymerase chain reaction (RT-PCR) is used to test for severe acute respiratory coronavirus virus 2 (SARS-CoV-2) in the specimens collected through swabs, as recommended by the World Health Organization for clinical management and outbreak control purposes.^3^ It is currently the gold standard for the etiological diagnosis of SARS-CoV-2 infection.

However, RT-PCR may fail to identify infected persons. A Chinese study of 610 hospitalized COVID-19 cases revealed that results of RT-PCR varied within the same patients throughout their diagnostic and therapeutic course and hypothesized a high rate of false-positive tests.^4^ False-positive tests were also suspected by Xiao et al^5^ in their study of 70 COVID-19 patients.

The University Hospital of Udine, Italy, serving a population of 530,000, has offered RT-PCR tests for detecting SARS-CoV-2 since the beginning of March, when the first COVID-19 case was suspected in the hospital catchment area. Swabs are collected from hospitalized or symptomatic persons, from asymptomatic close contacts of confirmed cases, identified through contact tracing, or for screening purposes. We investigated the possibility that a person with COVID-19 confirmed by a positive RT-PCR test on an upper respiratory specimen collected though swab had a subsequent false-negative test in the first 6 weeks of outbreak, analyzing the anonymous administrative database of the Virology Laboratory of the University Hospital of Udine, where subjects are identified by an anonymous univocal stochastic key. For patients with at least 1 positive test (COVID-19 cases), we assessed false-negative tests, defined as negative tests between 2 positive tests.

From March 1 to April 12, our laboratory processed 15,702 RT-PCT tests on 10,482 people, and we identified 860 new COVID-19 patients (Table 1). The daily number of exams increased progressively exceeding 1,000 by April 9, whereas the proportion of those resulting positive peaked on March 17 (23.5%) and then progressively decreased.

**Table 1.** Age Distribution of COVID-19 Cases Identified Through RT-PCR Test for SARS-COV-2 on Upper Respiratory Specimens Collected Through Nasal Swabs, University Hospital of Udine, Italy, Between March 1, 2020, and April 12, 2020

Age Group	COVID-19 Cases	Cases With at Least 2 Exams After the Positive Test, No. (%)	Cases with False-Negative Result of All With at Least 2 Additional Exams, No. (%)
0–14	11	7 (63.6)	3 (42.9)
15–44	207	138 (66.7)	26 (18.8)
45–64	280	187 (66.8)	37 (19.8)
65–74	120	58 (48.3)	13 (22.4)
75–89	162	37 (22.8)	3 (8.1)
≥90	80	6 (7.5)	2 (33.3)

Subjects with >1 swab collection were 2,949 (28.1%). The proportion increased from 25.1% among 9,658 subjects with initial negative exam, to 37.3% among 59 with initial invalid exam, to 65.9% among 765 with initial positive result (χ^2^ test, *P* < .0001). The median times from first to second exam were 7, 1, and 11 days, respectively.

Of 860 COVID-19 cases, 433 had at least 2 additional swabs after the first positive result. The likelihood of having at least 2 additional swabs decreased significantly among the elderly (χ^2^ test, *P* < .0001) (Table 1). Of COVID-19 cases with at least 2 additional exams, 84 (19.4%) had a negative result after the COVID-19 diagnosis, followed by a positive result. The proportion did not vary significantly across age groups (Fisher exact test, *P =* .1821) (Table 1). Among those 84 COVID-19 cases, median time from the negative swab and the following positive swab was 2 days. Only 2 persons had 1 positive result after 2 consecutive negative tests.

Negative RT-PCR tests followed, within few days, by a positive result among COVID-19 confirmed cases can be reasonably considered a false negative because the same patients had a positive test immediately afterward. Our population included both symptomatic and asymptomatic SARS-CoV-2 infections. Our results are important not only for hospitalized patients, who might be discharged based on false-negative results but also for asymptomatic cases who might break isolation based on tests that might by not reliable. If those persons are still infectious, they can spread the virus in the community.

Lippi et al^6^ described potential RT-PCR vulnerabilities that may affect the diagnostic accuracy of this technique, including both general preanalytical issues (collection, handling, transport and storage of the swabs, quality and volume of the collected material, interference from other substances) and analytical issues (choosing the right diagnostic window, validation of assays, harmonization, instrument functioning). Ways to minimize the risk of diagnostic errors include repeated collection of specimens in patients with suspicion of infection, training on swab collection, quality assurance for analytical procedures, and combination of clinical evidence with RT-PCR results.^6^ Laboratory parameters, such as lactate dehydrogenase, C-reactive protein, alanine aminotransferase, neutrophil count,^7^ and results of chest computed tomography^8^ can help define the disease stage.

We were able to assess only the proportion of false-negative tests among subjects with multiple swabs collected after a positive test. Conversely, if a subject had a negative test (either the first or another one) and no further swabs, it was not possible to assess whether the test was truly negative. Nonetheless, we assume that our results are generalizable to all tests. Thus, a first negative result should not be sufficient to neglect social distancing measures or use of personal protective equipment.

For a better understanding of the role and diagnostic accuracy of RT-PCR for SARS-CoV-2, further research should be conducted to assess viral load in respiratory specimens in patients with different severity of infection and at different time points.
